# Supplementary material for: A Comprehensive Analysis of Immune Response in Patients with Non-Muscle-Invasive Bladder Cancer
Source: Cancers (Basel). 2023 Feb 21;15(5):1364. doi: 10.3390/cancers15051364 (PMC10000243; doi:10.3390/cancers15051364)
Supplement: Supplementary file 1 [file cancers-15-01364-s001.zip › cancers-2217273-supplementary.pdf]

cancers

MDPI

Supplementary Material

Supplementary Table 1. Antibodies and fluorochromes

Panel	Antibodies	Fluorochrome	Provider
**Panel 1 (T lymphocytes 1)**	Mouse anti human CD49b	FITC	BD Biosciences
	Mouse anti human CD127	PE	BD Biosciences
	Mouse anti human CD3	PerCP	BD Biosciences
	Mouse anti human LAG3 (CD223)	Alexa Fluor 647	BD Biosciences
	Mouse anti human CD4	APC H7	BD Biosciences
	Mouse anti human CD25 (TP1/6.2)	(secondary Pacific blue)	D. Sánchez-Madrid
	Mouse anti human CD8	V 500	BD Biosciences
**Panel 2 (T lymphocytes 2)**	Mouse anti human TIM3	VioBright FITC	Miltenyi
	Mouse anti human CD279 (PD1)	PE	BD Biosciences
	Mouse anti human CD3	PerpCP	BD Biosciences
	Mouse anti human CD278 (ICOS)	Alexa 647	BD Biosciences
	Mouse anti human CD27	PeCy7	BD Biosciences
	Mouse anti human CD4	APC H7	BD Biosciences
	Mouse anti human CD8	V 500	BD Biosciences
**Panel 3 (myeloid cells)**	Mouse anti human MHCII	FITC	BD Biosciences
	Mouse anti human CD 127a/b (SIRP)	PE	BD Biosciences
	Mouse anti humanIDO1	Alexa 647	BD Biosciences
	Mouse anti human CD14	APC H7	BD Biosciences
	Mouse anti human CD11c	V450	BD Biosciences
	Mouse anti human CD123	BV 510	BD Biosciences
**Panel 4 (NK cells)**	Mouse anti human KIR	PE	BD biosciences
	Mouse anti human NKG2a	PerCp	R&D systems
	Mouse anti human NKG2d	PeCy7	BD biosciences
	Mouse anti human CD56	V 450	BD biosciences
